# Patient and family caregiver adaptation during hospital-home transition: a concept analysis

**DOI:** 10.1590/1980-220X-REEUSP-2024-0363en

**Published:** 2025-04-25

**Authors:** Alejandra Fuentes-Ramírez, Gloria Carvajal-Carrascal, Karen Tatiana Roa-Lizcano, Laura Ximena Peña-Mancera, Beatriz Sánchez-Herrera

**Affiliations:** 1 Universidad de La Sabana, Facultad de Enfermería y Rehabilitación, Chía, Colombia.

**Keywords:** Adaptation, Transitional Care, Patient Discharge, Caregivers, Nursing Theory, DeCS, Adaptação, Cuidado Transicional, Alta do Paciente, Cuidadores, Teoria de Enfermagem, DeCS

## Abstract

**Objective:**

To analyze the concept of “Adaptation of the patient and his family caregiver” during the hospital-home transition”.

**Method:**

The Walker and Avant method was followed. This included a scoping review following the Joanna Briggs Institute (JBI) parameters conducted in the Dimensions and Eureka metasearch engines and the PubMed, Embase, Scielo, CINAHL, and ScienceDirect databases.

**Results:**

Of 6073 articles, 85 met inclusion criteria. The transition from hospital to home of the patient and his/her family caregiver after discharge from the hospital requires them to take on care tasks for which they are not always prepared. Adapting to this transition involves understanding it, coping with it; having support; anticipating risks; transferring instructions; adhering to therapy; and monitoring and recording the health condition.

**Conclusion:**

The adaptation of the patient and his/her family caregiver during the hospital-home transition is a comprehensive response to the responsibility of caring for his/her health. Adapting means being able to reestablish routines, maintain or improve quality of life, strengthen autonomy and have a sense of achievement and control over the situation.

## RESUMO

**Objetivo:**

Analisar o conceito de “Adaptação do paciente e seu cuidador familiar durante a transição hospital-casa”.

**Método:**

O método Walker e Avant foi seguido. Isto incluiu uma revisão de escopo que seguiu os parâmetros do Joanna Briggs Institute (JBI), conduzida nos Mecanismos de metabusca Dimensions e Eureka e bancos de dados PubMed, Embase, Scielo, CINAHL e ScienceDirect.

**Resultados:**

De 6073 artigos, 85 atenderam aos critérios de inclusão. A transição hospital-casa do paciente e de seu cuidador familiar após a alta hospitalar exige que eles assumam tarefas de cuidado para as quais nem sempre estão preparados. Adaptar-se a essa transição envolve compreendê-la e enfrentá-la; ter suporte; prever riscos; transferir instruções; aderir à terapia; e monitorar e registrar as condições de saúde.

**Conclusão:**

A adaptação do paciente e do seu cuidador familiar durante a transição hospital-casa é uma resposta abrangente à responsabilidade de cuidar da sua saúde. Adaptar-se significa ser capaz de restabelecer rotinas, manter ou melhorar a qualidade de vida, fortalecer a autonomia e ter uma sensação de realização e controle sobre a situação.

## INTRODUCTION

The World Health Organization [WHO] proposes as one of the central strategies to guarantee patient safety, seeking safe transitions between different care settings such as the hospital and the patient’s home. According to WHO, continuity and coordination during this transition can reduce mortality, complications, hospital readmissions, and family suffering and burden^(1)^.

Health systems have sought to generate appropriate strategies so that patients and caregivers receive the required responses during the hospital-home transition^(2)^. These strategies include assessment, trust building, commitment strengthening, medication management, symptom recognition and management, education, self-care promotion, team-family collaboration, follow-up with calls and home visits, and adjustments in system resources and processes^(3, 4, 5, 6, 7, 8, 9, 10)^. In these programs and strategies, the role of nursing has proven to be particularly useful when performing care, administration, collaboration, guidance, education, and advocacy functions for the subjects under their care^(11, 12, 13, 14, 15)^.

Despite invaluable advances in the field of care during the hospital-home transition, reported studies reflect a lack of agreement on the terms and concepts related to people’s adaptation during this transition. This absence is reflected in services that have not evolved sufficiently to support users, nor to adequately coordinate institutional care with home care^(16)^. This also generates a lack of coordination or standardization of the processes or the skills required to team work, which in turn causes a greater perception of work overload and lack of time and resources^(8)^. The gap is also reflected in the fact that some hospital-to-home transition programs have been shown to reduce complications and hospital readmission of patients, while others have not shown good results or have failed to benefit different populations^(17, 18, 19, 20, 21)^.

To guide practice and research, the sharing and communication of data and findings and the creation of common guidelines based on the best evidence to support care are required. To enhance the adaptation of patients and their family caregivers during the hospital-home transition, this knowledge gap shall be filled. In this regard, this work sought to specify this concept’s attributes, antecedents, and consequences as a contribution to health care in this field.

## METHOD

This is a concept analysis study including the eight steps proposed by Walker and Avant^(22)^. This method seeks to clarify the meaning of a concept to facilitate a common language that can guide research and practice in care, in this case during the hospital-home transition, and that supports theoretical development in the field. This method consists of eight steps, including: selecting the concept; establishing the objective of the analysis; identifying uses of the concept; determining its attributes; identifying model cases and additional cases that illustrate it; defining its antecedents and consequences; and defining its empirical indicators^(22)^.

The definitions of the terms of the concept “Adaptation of the patient and his/her family caregiver during the hospital-home transition” were sought in dictionaries. A scoping review was conducted as it helps to clarify and define the key concepts related to the topic of study, allowing an overview of the available research with its different perspectives and approaches. The steps included: identifying the guiding question for the review; identifying sources of prior information; defining inclusion and exclusion criteria; conducting an exhaustive literature search to identify relevant studies; selecting these studies; extracting relevant data and contributions to the present research; analyzing them for emerging patterns and themes; and presenting the findings. To ensure its methodological rigor, this review followed the criteria by Khalil et al.^(23)^ under JBI approaches. To obtain the information, the metasearch engines Dimensions and Eureka and the databases PubMed, Embase, Scielo, CINAHL, and ScienceDirect were used. No limits were set on time, language, or location of the study. Grey literature, studies that did not address the concept of patient, caregiver, or context adaptation during the hospital-home transition, those that were not peer-reviewed, and those whose full text was not available were excluded. The search protocol was registered on the Open Science Framework (OFS) platform^(24)^.

Based on the search results, the attributes of the concept were reviewed and the cases were identified: the model case to reflect the attributes and consequences of the concept; the borderline case, where not all the conditions for the adaptation of the patient and his family caregiver during this transition are met; and the contrary case to illustrate the maladaptation in the process. Subsequently, the antecedents and consequences of this concept were analyzed and the proposed empirical indicators were identified to assess the adaptation of the patient and his/her family caregiver during the hospital-home transition.

## RESULTS

The analysis of the concept “Adaptation of the patient and his/her family caregiver during the hospital-home transition” responds to the lack of clarity on the subject in the literature and the need to specify it to develop programs promoting this adaptation.

### Definition of Terms of the Concept

The term adaptation means understanding the action or process of change to adjust to a new purpose or situation^(25)^. Adaptation can be internally or externally motivated, and includes cognitive and evaluative elements; it is associated with changes in attitude or behavior necessary for survival^(26)^. All living beings have functional adaptation processes that allow them to adapt to their environment^(27)^. Psychology reveals that adaptation arises from stress derived from changes in roles or contexts^(28)^. Pedagogy proposes that adaptation occurs when a person changes place, status, or condition in their roles, relationships, routines, or self-concept and that this requires perceiving situations and being aware of one’s own ability to confront them^(29)^.

The term transition refers to the process of moving from one situation or condition to a different one. This term can be applied to a variety of contexts. In the political or economic field it can refer to a change of regime or system^(30)^. When referring to transition in the health field, transitional care is defined as care that occurs where the patient and his or her family caregiver are physically or virtually present. This includes the transition between the hospital and home^(31)^.

The patient is the one who seeks or receives health care to improve his or her well-being, prevent or manage illnesses or injuries, or to obtain a diagnosis of his or her condition^(32)^.

A family caregiver is a person with a family or close relationship who assists someone who has limitations in their physical, mental or cognitive functioning, generally without receiving remuneration. As a group, they vary in age, location, and type of care they provide, as well as motivation and competence to provide such care^(33)^.

### Literature Review

**Figure 1:**
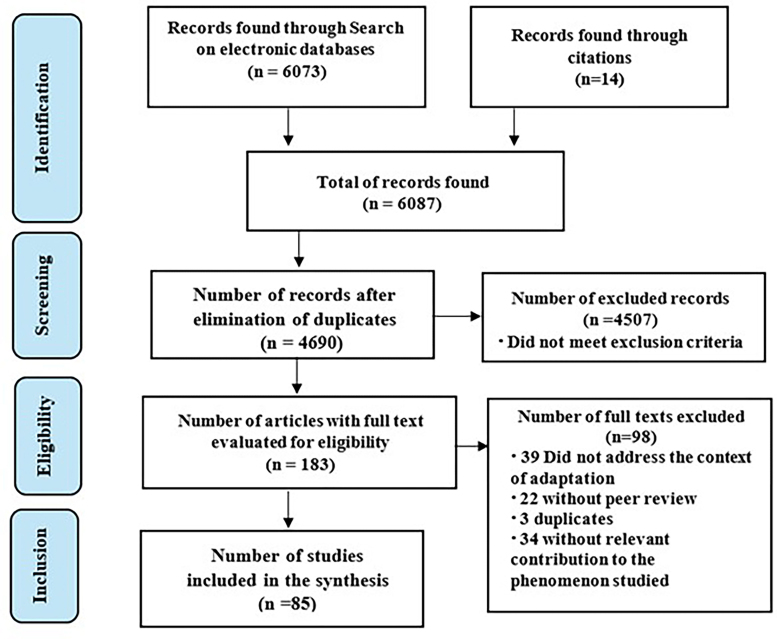
Flowchart for the selection and inclusion of studies (adapted from the PRISMA statement for publication of scoping reviews)

Source: Elaborated by the authors.

The literature review reported 6073 documents of which 85 were analyzed, as presented in Figure 1.

### Characteristics and Attributes of Patient and Family Caregiver Adaptation During the Hospital-Home Transition

The transition from hospital to home is a complex process that varies according to the situation of the subjects involved, the condition they face, and the context in which they do so; this process is characterized by generating high levels of uncertainty and stress for the patient and their family caregiver and requiring significant adjustment^(34, 35)^. Adaption during this transition is to achieve the best possible quality of life for the patient and their family caregiver, avoiding complications and strengthening autonomy until achieving the integration of care into daily routine^(36)^; it is taking control and regaining a new normal^(37, 38, 39, 40, 41, 42, 43)^. This adaptation includes seven attributes:

***Coping.*** For the patient-family caregiver dyad, coping is a way of accepting one’s own reality, recognizing oneself, and assuming responsibility for care during the transition, feeling themselves as its active parts^(44, 45, 46)^. It is a difficult internal process for those who live in highly vulnerable situations, but adaptation is necessary^(41, 47, 48)^. It is the acceptance of the new reality with the decision to assume responsibility for care^(49, 50)^.

***Discerning*.** The patient and their family caregiver have to discern their situation so that they can address it^(41, 51, 52, 53, 54)^. That is, knowing, understanding, and being able to differentiate aspects related to their condition and health care during the transition from the hospital to home^(50, 55, 56, 57, 58)^. Understanding their own situation to be able to manage it until achieving autonomy^(55, 57, 59, 60, 61, 62, 63, 64)^.

***Having support.*** Successful adaptation of patients and their caregivers during the transition from hospital to home requires having the necessary support^(56, 65, 66)^. This support may be associated with information, preparation for discharge or for aftercare^(44, 67)^. It may also refer to the need to guarantee home and community care services for the dyad^(68)^. The need for support must be identified by the subjects of care based on the recognition of the gaps to adopt the care required and to face changes in functionality or routine^(34, 39, 41, 57, 59, 69)^. Likewise, the dyad must recognize the necessary and available resources to deal with the situation^(70, 71, 72)^. The required support includes social support, provided by family and friends, personal or peer networks; professional support, with adequate accompaniment or supervision and advanced information; emotional or spiritual support to adequately cope with the process; operational support to aid in care tasks; and system support to ensure the necessary resources and special supplies in the health care situation during the hospital-home transition^(49, 50, 51, 53, 55, 58, 62, 65, 73)^. Support may fluctuate during the different stages of transition, with those required after hospital discharge being of particular interest to the patient and their family caregiver^(48)^.

***Preventing*.** Adaptation in the transition process requires prevention to ensure safety during care tasks, identification of risks, and reduction of the likelihood of them being materialized^(49, 51, 58, 71)^. Asking for guidance, assistance, or timely help^(57, 58, 65)^. Knowing who to call and when to address potential health condition issues or to handle emergencies that may arise at home^(41, 44, 61)^.

***Transferring*.** Patients and their caregivers should transfer health care instructions to practices to respond in the best possible way to their health care during the hospital-home transition^(43, 54, 55, 56, 61)^. This includes the knowledge and skills to handle activities of daily living, basic and specialized care, treatment, and therapies^(41, 53, 62, 69, 74)^. When putting the instructions into practice, people have new doubts that must be resolved over time^(51, 61)^.

***Adhering.*** Adhering in this adaptation process means that the dyad is committed to following the guidelines prescribed for the patient’s condition over time and in the context in which they find themselves^(65)^. It is to handle the indicated therapy, attending to and following medical prescriptions based on drug reconciliation^(47, 62, 71)^. This may involve having to modify daily behavior to comply with prescribed instructions^(55, 70)^.

***Monitoring and keeping track*.** Adaptation during the hospital-home transition involves being attentive to the health condition, the effect of treatment and the response to care, as well as keeping a record of the evolution and its management with symptom monitoring, management during daily activities, and reporting changes or adjustments^(51, 58, 63, 75)^.

### Cases that Illustrate the Concept

***Model case***. Mrs. Anita is an 88-year-old widow with intact functionality and cognition, who lived with her daughter. Anita was admitted to the hospital with a broken ankle and returned home after being discharged. Her daughter was willing to face the situation and take care of her (coping). The daughter asked health professionals to explain her mother’s condition and how she should care for her, which allowed her to better understand the situation (discerning). She then received nursing support through written and verbal instructions during hospital discharge (having support). For three weeks, she had to keep the affected leg elevated to avoid swelling (preventing) and care for the wound twice a day, thus following the instructions given by the doctor (transferring). After 20 days, Anita began her rehabilitation and started reorganizing things together with her daughter, while adhering to the instructions and exercises for healing. The usual pharmacological treatments and rehabilitation exercises were then continued as recommended (adhering). When going to the follow-up appointment, Anita and her daughter presented the completed records of the exercises performed and weekly blood pressure (monitoring and recording). These records allowed for a small adjustment to be made to his medication. Today, Anita and her daughter have resumed their normal routines. Things seem to be back to normal, although since her accident they have learned to be more attentive to prevent situations that could cause other falls.

***Borderline case.*** Camilo and María are 43 and 40 years old respectively, they have been married for 10 years and decided not to have children. They feel that their mutual support has been decisive in overcoming difficulties. Camilo left the hospital after starting his hemodialysis treatment and will have to return twice a week for the rest of his life or go to the peritoneal dialysis program until they find him a donor. The medical staff have explained him that this situation will not change and although Camilo understands this, María does not believe what they tell her; she hopes that Camilo’s kidney function will be restored so that he can continue with his daily activities. She says she knows of cases of urinary problems that have improved on their own. They hired an emergency service for any unforeseen event based on the fear of the situation. María and Camilo follow the food care instructions when preparing the daily menu, except for small bites that they do not include in the food record that they must take to the medical check-up “so that the health personnel do not scold us.”

***Contrary case***. Juan is a delivery man who presented with abdominal pain. He has state-subsidized social security and lives with Marta, his wife, and their one-year-old twin sons. He went to the emergency room and was told he had appendicitis and needed urgent surgery. Marta asked a neighbor for help to take care of her children while she helped Juan in the hospital, but there were complications and Juan had to stay alone in the surgery department for days. Marta could only enter during visiting hours, but in this period the neighbor could not help her take care of the twins. Juan said that she abandoned him and wanted to run away. He does not know how to handle the situation. He did not ask for help at the hospital because he thought they would not give it. Eventually, Juan left the hospital and ignored instructions for his care, such as taking medication and resting; he did not know how to ask for his medicines. Eight days later, he had to be admitted again due to infection. The situation was overwhelming for Juan and Marta and changed their relationship significantly.

### Identification of Antecedents and Consequences

***Antecedents***. Adaptation during the hospital-home transition of the patient-family caregiver dyad requires that they be ready for discharge, that they have guidance and support during this period, that they understand and get involved in the care, resolving their doubts and guaranteeing follow-up^(63, 65, 68)^. This adaptation demands that they feel recognized and respected as people and that the enhancement of their care capabilities is encouraged by a comprehensive look at them^(76, 77)^. Of particular relevance in this process are adequate information, communication, and coordination before hospital discharge^(61, 67, 70, 78, 79, 80, 81)^ as well as the review and adjustment of the context conditions and the coverage of health care needs^(69, 82)^.

This dyad has to recognize their condition and their abilities to successfully face the challenge of care in the midst of ignorance, fear and vulnerability but with the desire to move forward seeking to reduce the burden and achieve well-being in daily life^(49, 56, 64, 70, 71, 74, 83, 84)^. It implies that the dyad can meet care needs during the process to face barriers and take advantage of opportunities^(72, 85)^. Those involved have to seek control and a state of harmony to learn how to manage a new health condition that is added to the usual demands^(16, 46, 50, 55, 65, 86)^. It is assumed that they generate positive coping strategies that help them perceive their ability to care, to reduce stress or anxiety, to strengthen their potential, their resources and their mutual bond and to recognize their limitations and increase their level of resilience^(34, 38, 52, 54, 57, 62, 63, 74, 78, 84, 87, 88, 89, 90, 91)^. To cope with it, the dyad needs to manage the conflict and address grief^(49)^, as well as have motivation and guidance from the hospital to take care of the patient at home^(51, 60, 61, 64, 67, 69, 70, 85, 86)^.

Achieving the discernment required to be able to under-stand and differentiate the situation and the care needed is a process that takes time and requires specific guidance and education regarding care tasks, while growing with experience^(34, 38, 45, 46, 58, 62, 70, 72, 73)^.

Getting effective support requires that the dyad accepts it as useful and reliable^(44, 73, 87)^. Professionals and institutions must recognize people in their current situation, guide them appropriately, coordinate processes and services, explain the care plan in detail, confirm good performance and motivate them to get involved to achieve the best possible results during the hospital-home transition^(16, 35, 38, 40, 41, 42, 43, 45, 46, 47, 50, 51, 52, 53, 54, 58, 59, 60, 61, 63, 64, 68, 78, 91, 92, 93)^.

Prevention requires knowing in advance the needs or problems that may arise and preparing to solve them or to minimize the risks during and after the transition^(40, 42, 45, 46, 71, 74, 92)^.

When leaving the hospital, the dyad requires preparation and support since they receive greater responsibility for care and must attend to and apply the instructions received^(45, 50, 53, 56, 58, 71)^. In this sense, it is necessary for professionals to verify their level of understanding and ability to assume this responsibility before discharge^(59, 64)^. Having clinical guidelines or protocols facilitates quality processes and helps prevent unnecessary complications^(16, 67)^.

Adherence is affected by the practical realities of maintaining treatment and by the inner disposition to do so; achieving good adherence implies having independence and credibility in those who guide treatment and care to do what should be done and avoid what should not be done^(90)^.

Finally, monitoring the health condition by maintaining constant follow-up, recording relevant findings, is necessary to help in decision-making and to receive better guidance for managing the condition^(41, 47, 62, 71)^. Proper registration prevents omissions and makes it easier to specify the needs and support required during professional follow-ups^(64)^. The use of technology and the prior assessment of its relevance can facilitate them^(94, 95)^.

***Consequences.*** Those who adapt themselves during the hospital-home transition manage to regain control of the situation with greater autonomy in managing care tasks. They can achieve a new normal with personal satisfaction, confidence and comfort by integrating care into their daily routine. They can glimpse the future from the new condition and establish a life plan where worry and anxiety decrease, overcoming obstacles to integrate themselves into their own community^(44, 51, 54, 57, 59, 62, 71, 73, 75, 88, 90, 91, 92, 93)^.

The adaptation of the patient and his/her family caregiver during the hospital-home transition reflects adequate coping that generates confidence, self-control, and self-regulation and allows for the organization of care routines and tasks^(50, 51, 53, 55, 56, 71)^. This generates a positive perception with a sense of competence and control in the midst of an altered routine^(16, 35, 40, 42, 53, 54, 56, 58, 59, 80)^.

Similarly, adaptation is associated with a good level of discernment, which allows the dyad to understand and be able to manage all the care required with a positive impact on the health situation in their daily lives^(37, 49, 70, 71, 74)^.

Adequate adaptation of the patient and his family caregiver during the hospital-home transition involves having identified or built a support network that facilitates the experience^(63, 74)^. It reflects that professionals and institutions have provided adequate resources and have followed up on their fluctuating needs to respond to their requirements^(49, 55, 70, 71, 85, 86)^.

When dyads get adapted in this process, the existence of a care plan is also evident, in which personal and environmental precautions are taken and emergency or alarm signs associated with the health condition are identified^(51, 57, 62)^. Prevention helps them improve their quality of life and avoids complications, unnecessary expenses, hospital readmissions, and the unnecessary use of specialized services^(52)^.

Although therapeutic tasks or goals may be initially complex, they become part of a daily routine over time^(50, 65, 73)^. However, to achieve this, it is necessary for the patient and his family caregiver to ask and get involved in the care as much as possible^(43, 58)^. It is also necessary to provide the resources, adaptations, equipment, and information necessary to ensure the care required by these people at home^(52)^. Assuming the transfer by instrumentalizing care instructions appropriately allows the dyad to develop autonomy and increase the level of competence^(73)^.

Adherence has proven to be a practice that helps overcome critical health situations and generate a better life prognosis^(41, 46, 59)^; it also helps reduce risks and improve the effect of treatments that begin in the hospital and continue at home^(67)^. A good level of adherence to treatment is a condition that reflects that there is an adaptation process for care^(55)^.

Likewise, recording allows observing treatment progression and effect more objectively, and the acquisition of skills for care in the dyad, helping to specify their level of adaptation during the hospital-home transition^(72)^.

**Figure 2:**
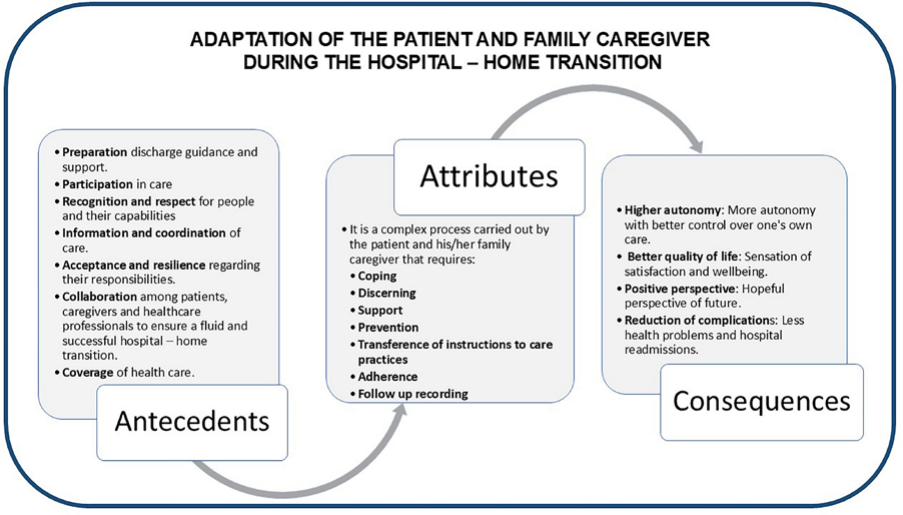
Adaptation of the patient and his family caregiver during the hospital-home transition

Source: Elaborated by the authors.

The result of a positive adaptation to caregiving allows for a reduction in perceived stress, maintaining a better quality of life, improving mood and satisfaction with one’s own performance in caregiving^(45, 52, 58, 87, 89, 94)^. Conversely, lack of adaptation is associated with greater complications and costs^(66)^ and increases the gap between the theory and practice of health care^(47)^. When this happens, it is necessary to find what makes adaptation difficult, what people would like to be different and what can help them in their process^(60)^. Figure 2 illustrates the antecedents, attributes, and consequences of the concept “adaptation during the hospital-home transition” (see Figure 2).

### Definition of Empirical Indicators

**Figure 3:**
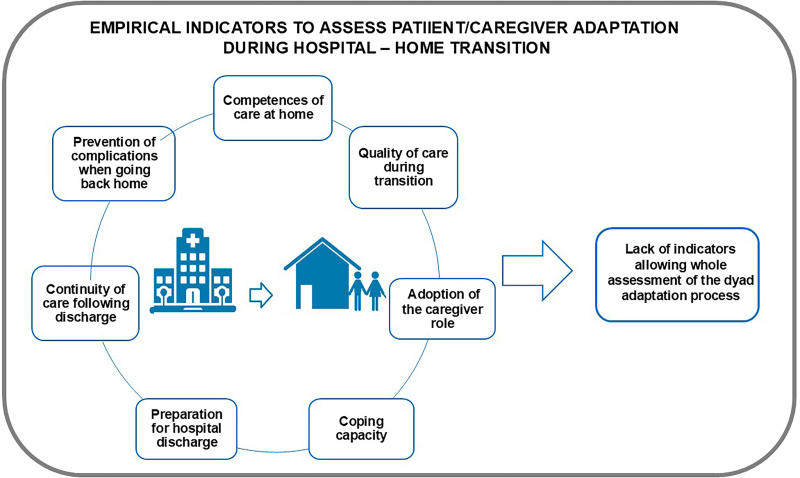
Empirical indicators of the adaptation of the patient and his family caregiver during the hospital-home transition

Source: Elaborated by the authors.

The adaptation of the patient-family caregiver dyad during the hospital-home transition has been assessed in different ways. The literature reports instruments applicable to this adaptation that evaluate home care competencies^(71, 96)^; quality of care during the transition^(97, 98, 99, 100)^; adoption of the caregiver role^(101)^; adoption of the ability to care^(102)^; coping ability^(103)^; preparation and readiness for hospital discharge where the health condition and the level of understanding of the condition are reviewed; the ability to cope and the level of support available^(104, 105)^; continuity of care after discharge^(106)^; prevention of unnecessary complications such as medication management upon returning home^(107)^. Some of these tools have been culturally validated^(108)^. However, none of these indicators comprehensively addresses the adaptation of the hospitalized person-family caregiver dyad during the hospital-home transition^(109, 110, 111)^ (See Figure 3).

## DISCUSSION

This research explored, described, and understood the adaptation, during the hospital-home transition, of the patient and his family caregiver, seeing them as a dyad^(34, 91)^. During the transition, the members of this dyad maintain a constantly evolving relationship with reciprocal influence and individual and joint needs that must be met to achieve a common goal^(39)^. On the one hand, the caregiver is at risk of perceiving physical and psychological burden and seeing their daily life affected. On the other hand, the patient must accept his situation and seek optimal clinical results. Comprehensive care, which is necessary during the hospital-home transition, must consider the needs and dynamics of the dyad to achieve their adaptation to the required care^(40, 57, 68, 101)^.

The analysis of the concept “adaptation of the hospitalized person-family caregiver dyad during hospital-home transition” is a contribution to current knowledge. On the one hand, it adds some concepts related to this topic, such as the concept of transitions for nursing^(112)^; the concept of patient-centered care with empowerment and participation in hospital care^(113)^; the concept of person-centered care^(114)^; peer support in the context of health care^(115)^; and the concept of transitional care at the level of health systems^(116)^. It also complements the models and theories related to this topic, such as the model for the analysis of adaptation to human transitions^(117)^ and care models during the transition^(118)^.

From the theoretical perspective of nursing, the concept of “adaptation of the patient and his family caregiver during hospital-home transition” is consistent with the postulates of the Callista Roy Adaptation Model and the middle-range theories developed from it, which propose how the nurse should facilitate the patient’s adaptation to promote their health and well-being^(119, 120)^. Regarding the theory of transitions proposed by Meleis et al. ^(121)^, which includes those experienced by individuals throughout their lives, those that occur between health and illness, those that occur with age, and those that are associated with other situations of change, this concept includes, as its author points out, the context, emotions, and coping strategies of the patient and their family caregiver since they can affect the adaptive process during the transition^(122)^. The present study confirms the arguments by Ribeiro et al.^(123)^, regarding the important level of coherence between the theoretical conceptions of Callista Roy and those of Afaf Meleis, and their application in professional practice. The concept analyzed reflects that adaptation is an end and transition is the changing scenario where the health care process is developed by the patient-family caregiver dyad. These approaches reflect the analysis presented by Meleis, who proposes the evolution of nursing from large to medium theories to address more specific situations of practice^(124)^.

These findings may be useful to policy makers and nursing educators and assistants as a basis for developing evidence-based interventions to improve assessment, diagnosis, and outcomes during the hospital and discharge transition of patients and their family caregivers. Conceptual clarity shows to be, as it has been documented, an essential element of nursing practice^(125)^. The present analysis was approached with inclusive criteria in geography and languages. However, their findings may have limitations in light of some specific hospital-home transition practices not yet reported in the literature. Similarly, there may be restrictions for those who seek to understand the phenomenon studied from the individual perspective of the patient or their caregiver without considering them as a dyad.

## CONCLUSION

Adaptation during the hospital-home transition is a desirable event that can occur at various levels and periods. These begin when patients and their family caregivers enter the hospital and last until they are able to care for their health. Adaptation during the hospital-home transition is a reflection of the best decisions for patients and caregivers to maintain or improve their quality of life at home.

This transition creates changes and challenges for patients and their family caregivers and is mediated by the way they cope with situations, how they discern the information available to them, the support they have, the prevention and anticipation of risk and emergencies, their ability to translate therapeutic instructions into actions, their adherence to care and treatment instructions and monitoring, and recording to facilitate decision-making.

Adaptation during the hospital-home transition of these dyads is a challenge for nursing, who, by understanding both the vulnerability and the care potential of patients and their family caregivers, will be able to support the achievement of a better quality of life with fewer complications and care burden.

The definition, attributes, antecedents, and consequences of adaptation during the hospital-home transition obtained in this study provide a theoretical basis for future research. This information can be used to assess adaptation during the hospital-home transition, develop assessment tools, and generate theory-based training and interventions.
