# Supplementary material for: Patient and family caregiver adaptation during hospital-home transition: a concept analysis
Source: Rev Esc Enferm USP. 2025 Apr 25;59:e20240363. doi: 10.1590/1980-220X-REEUSP-2024-0363en (PMC12024360; doi:10.1590/1980-220X-REEUSP-2024-0363en)
Supplement: Translation

# Translation: Adaptación del paciente y su cuidador familiar durante la transición hospital-hogar: análisis de concepto

**DOI:** 10.1590/1980-220X-REEUSP-2024-0363es

**Authors:** Alejandra Fuentes-Ramírez, Gloria Carvajal-Carrascal, Karen Tatiana Roa-Lizcano, Laura Ximena Peña-Mancera, Beatriz Sánchez-Herrera

**Affiliations:** 1 Universidad de La Sabana, Facultad de Enfermería y Rehabilitación, Chía, Colombia.

**Keywords:** Adaptación, Cuidado de Transición, Alta del Paciente, Cuidadores, Teoría de Enfermería, DeCS

## Abstract

**Objetivo:**

Analizar el concepto “Adaptación del paciente y su cuidador familiar durante la transición hospital-hogar”.

**Método:**

Análisis bajo lineamientos de Walker y Avant con revisión de alcance. Se siguieron parámetros del Johanna Brinings Institute, usando los metabuscadores Dimensions y Eureka y las bases de datos PubMed, Embase, Scielo, CINAHL y ScienceDirect.

**Resultados:**

85 artículos cumplieron criterios de inclusión para ser revisados. La transición hospital - hogar inicia con la hospitalización de un paciente quien regresa a su hogar junto con su cuidador familiar después del alta hospitalaria. Allí deben asumir tareas de cuidado para las cuales no siempre están preparados. Su adaptación les implica comprender y afrontar la situación; buscar apoyo; anticiparse a riesgos; transferir instrucciones; adherirse a un plan terapéutico; y monitorear y registrar la condición de salud.

**Conclusión:**

La adaptación del paciente y su cuidador familiar durante la transición hospital-hogar, es una respuesta integral frente a la responsabilidad de cuidar su salud. Adaptarse significa reestablecer rutinas, mantener o mejorar la calidad de vida, fortalecer la autonomía y tener sensación de logro y control de la situación.

## INTRODUCCIÓN

La Organización Mundial de la Salud [OMS] (2024), propone como una de las estrategias centrales para garantizar la seguridad de los pacientes, buscar transiciones seguras entre los diferentes escenarios de cuidado como son el hospital y el hogar. Según ellos, la continuidad y la coordinación durante esta transición pueden disminuir la mortalidad, las complicaciones, los reingresos hospitalarios y el sufrimiento y carga familiar^(1)^.

Los sistemas de salud han buscado generar estrategias adecuadas para que los pacientes y cuidadores reciban las respuestas requeridas durante la transición hospital – hogar^(2)^. Estas estrategias abarcan la valoración, la construcción de confianza, el afianzamiento del compromiso, el manejo de medicamentos, el reconocimiento y manejo de los síntomas, la educación, la promoción del autocuidado, la colaboración entre el equipo y la familia, el seguimiento con llamadas y visitas domiciliarias y los ajustes en recursos y procesos de los sistemas^(3,4,5, 6, 7, 8, 9, 10)^. En estos programas y estrategias, el rol de enfermería ha demostrado ser particularmente útil cuando se ejercen funciones asistenciales, de administración, colaboración, orientación, educación y abogacía por los sujetos a su cargo^(11, 12, 13, 14, 15)^.

A pesar de los invaluables avances en el campo del cuidado durante la transición hospital hogar, algunos servicios no han evolucionado lo suficiente para apoyar a los usuarios y para coordinar de manera adecuada la atención institucional con la domiciliaria^(16)^. Se ha documentado que, para su funcionamiento apropiado, aún deben superar dificultades como la sobrecarga de trabajo, la falta de tiempo de los profesionales, de recursos, de procesos coordinados y estandarizados y de competencias para trabajar en equipo^(8)^. En algunos casos los programas de transición hospital - hogar han demostrado disminuir las complicaciones y la readmisión hospitalaria de los paciente^(17, 18, 19)^, en otros, no se han logrado mejores resultados^(20)^ o su diseño no es equitativo para beneficiar a diferentes poblaciones^(21)^.

Los estudios reportados reflejan un vacío de conocimiento respecto a la adaptación del paciente y su cuidador familiar durante la transición hospital - hogar. En tal sentido el presente trabajo buscó precisar los atributos, antecedentes y consecuencias de este concepto como un aporte que apoye el mejoramiento de las prácticas de salud en este campo.

## MÉTODO

Este es un estudio de análisis de concepto que incluye los ocho pasos propuestos por Walker y Avant^(22)^. Se buscaron inicialmente las definiciones de los términos del concepto “Adaptación del paciente y su cuidador familiar durante la transición hospital-hogar” en diccionarios. Se realizó una revisión de alcance para revisar su uso en la literatura. Para garantizar su rigor metodológico esta revisión siguió los criterios de Khalil et al.^(23)^ bajo planteamientos del Johanna Brinings Institute (JBI). Para obtener la información se emplearon los metabuscadores Dimensions y Eureka y las bases de datos PubMed, Embase, Scielo, CINAHL y ScienceDirect. No se establecieron límites de tiempo, idioma o lugar del estudio. Se excluyeron la literatura gris, los estudios que no abordaran el concepto de adaptación del paciente, su cuidador o su contexto durante la transición hospital - hogar, los que no fueran evaluados por pares y aquellos cuyo texto completo no estuviera disponible. El protocolo de la búsqueda fue registrado en la plataforma Open Science Framework (OFS)^(24)^.

A partir de los resultados de la búsqueda se revisaron los atributos del concepto y se identificaron los casos: el caso modelo para reflejar los atributos y consecuencias del concepto; el límite donde no se dan todas las condiciones para la adaptación del paciente y su cuidador familiar durante esta transición y el caso opuesto para ilustrar la desadaptación en el proceso. Posteriormente, se analizaron los antecedentes y consecuencias de este concepto y se identificaron los indicadores empíricos propuestos para valorar la adaptación del paciente y su cuidador familiar durante la transición hospital-hogar.

## RESULTADOS

El análisis del concepto “Adaptación del paciente y su cuidador familiar durante la transición hospital-hogar” responde a la falta de claridad sobre el mismo en la literatura y a la necesidad de precisarlo para poder desarrollar programas que promuevan esta adaptación.

### Definición de Términos del Concepto

El término adaptación significa comprender la acción o proceso de cambio para ajustarse a un nuevo propósito o situación^(25)^. La adaptación puede ser motivada interna o externamente, e incluye elementos cognitivos y evaluativos; se asocia con cambios de actitud o comportamiento necesarios para la supervivencia^(26)^. Todos los seres vivos tienen procesos de adaptación funcional que les permite acomodarse a su entorno^(27)^. La psicología revela que la adaptación surge del estrés derivado de cambios de roles o contextos^(28)^. La pedagogía plantea que la adaptación ocurre cuando una persona cambia de lugar, estatus o condición en sus roles, relaciones, rutinas o autoconcepto y que esta requiere percibir las situaciones y ser consciente de la propia capacidad para afrontarlas^(29)^.

El término transición se refiere al proceso de pasar de una situación o condición a otra diferente. Este término puede aplicarse a diversos contextos. En el campo político o económico puede referirse a un cambio de régimen o de sistema como la transición de dictadura a la democracia o de feudalismo al capitalismo^(30)^. Al referirse a la transición en el campo de la salud, se habla de cuidado transicional como aquel que se da en donde el paciente y su cuidador familiar se encuentren físicamente o desde donde haga contacto con el profesional de la salud. Esto incluye la transición entre el hospital y el hogar^(31)^.

El paciente es quien busca o recibe atención de salud para mejorar su bienestar, prevenir o manejar enfermedades o lesiones o para obtener el diagnóstico de su condición^(32)^.

El cuidador familiar es una persona con vínculo de parentesco o cercanía que asiste a alguien que tiene limitaciones en su funcionamiento físico, mental o cognitivo, generalmente sin recibir remuneración. Como grupo varían en edades, ubicaciones y tipo de cuidado que ofrecen, así como en la motivación y la competencia para dar dicho cuidado^(33)^.

### Revisión de la Literatura

La revisión de la literatura reportó 6073 documentos de los cuales 85 fueron analizados, tal como se presenta en la Figura 1.

**Figura 1:** Flujograma para la selección e inclusión de los estudios (adaptado de la declaración PRISMA para publicación de revisiones de alcance)

Fuente, elaboración propia.

### Características y Atributos de la Adaptación del Paciente y su Cuidador Familiar Durante la Transición Hospital-Hogar

La transición hospital - hogar es un proceso complejo que varía de acuerdo con la situación de los sujetos involucrados, la condición que enfrenten y el contexto en que lo hagan; este proceso se caracteriza por generar al paciente y a su cuidador familiar altos niveles de incertidumbre y estrés y exigirles un ajuste significativo^(34, 35)^. Adaptarse durante esta transición es lograr la mejor calidad de vida posible para el paciente y su cuidador familiar, evitando complicaciones innecesarias y fortaleciendo la autonomía hasta lograr integrar los cuidados a la rutina diaria^(36)^; Es tomar el control y recobrar una nueva normalidad^(37, 38, 39, 40, 41, 42, 43)^. Esta adaptación incluye siete atributos:

***Afrontar***. Para la diada paciente cuidador familiar, afrontar es una forma de aceptar la propia realidad, reconocerse y asumir la responsabilidad del cuidado durante la transición sintiéndose parte activa de ella^(44, 45, 46)^. Es un proceso interno difícil para quienes viven situaciones de alta vulnerabilidad pero necesario para lograr la adaptación^(41, 47, 48)^. Es además lograr la aceptación activa de la nueva realidad con la decisión de asumir la responsabilidad del cuidado^(49, 50)^.

***Discernir***. El paciente y su cuidador familiar necesitan discernir su situación para poder abordarla^(41, 51, 52, 53, 54)^. Esto involucra conocer, comprender y ser capaz de diferenciar aspectos relacionados con su condición y cuidado de la salud durante la transición del hospital al hogar^(50, 55, 56, 57, 58)^. Es tomar conciencia de la propia situación, comprenderla para poderla manejar hasta lograr autonomía^(55, 57, 59, 60, 61, 62, 63, 64)^.

***Apoyarse***. Una adaptación exitosa de los pacientes y sus cuidadores durante la transición del hospital al hogar demanda contar con el apoyo necesario^(56, 65, 66)^. Este apoyo puede asociarse con la información, la preparación para el alta o para los cuidados posteriores^(44, 67)^. También puede hacer referencia a la necesidad de garantizar servicios de atención domiciliaria y comunitaria para el paciente y su cuidador familiar^(68)^. La necesidad de apoyo debe ser identificada por la diada a partir de reconocer los vacíos para poder asumir el propio cuidado, para enfrentar cambios en la funcionalidad o la rutina o para asumir cuidados especializados^(34, 39, 41, 57, 59, 69)^. De igual forma la diada debe reconocer los recursos necesarios y los disponibles para enfrentar la situación^(70, 71, 72)^. Los apoyos requeridos incluyen el social, brindado por familiares y amigos, redes personales o de pares; el profesional, con acompañamiento o supervisión adecuada e información avanzada; el emocional o espiritual para afrontar de manera adecuada el proceso; el operativo para apoyar las tareas de cuidado; y el del sistema para garantizar los recursos necesarios y los suministros especiales en la situación de cuidado de la salud durante la transición hospital hogar^(49, 50, 51, 53, 55, 58, 62, 65, 73)^. Los apoyos pueden además fluctuar durante las diferentes etapas de la transición siendo de especial interés para el paciente y su cuidador familiar aquellas que se presentan después del alta hospitalaria^(48)^.

***Prevenir***. La adaptación en el proceso de transición requiere prevenir para garantizar la seguridad durante las tareas de cuidado, identificar riesgos y reducir la probabilidad de que se materialicen^(49, 51, 71)^. Prevenir significa prepararse para identificar problemas que generan mayor vulnerabilidad como la posibilidad de infección cuando el sistema inmunológico está comprometido^(58)^. Pedir orientación, asistencia o ayuda oportuna^(57, 58, 65)^. Saber a quién y cuándo se llama para atender posibles problemas de la condición de salud o para manejar las posibles emergencias que se puedan llegar a presentar en el hogar^(41, 44, 61)^.

***Transferir***. Los pacientes y sus cuidadores deben transferir las instrucciones de atención sanitaria a las prácticas para responder de la mejor forma posible al cuidado de su salud durante la transición hospital – hogar ^(43, 54, 55, 56)^. Esta transferencia encierra los conocimientos y habilidades para atender tanto las actividades de la vida diaria y los cuidados básicos como los especializados, incluyendo el tratamiento, las terapias y el plan de cuidado^(41, 53, 62, 69, 74)^. Al llevar las instrucciones a la práctica, las personas tienen nuevas dudas que se deben ir resolviendo con el tiempo^(51, 61)^.

***Adherirse.*** La adherencia en este proceso de adaptación significa que la diada se comprometa a seguir las pautas prescritas para la condición del paciente a través del tiempo y en el contexto en que se encuentre^(65)^. Es manejar de manera adecuada la terapéutica indicada atendiendo y siguiendo las prescripciones médicas a partir de la reconciliación medicamentosa^(47, 62, 71)^. Esto puede implicar tener que modificar la conducta diaria para cumplir las indicaciones prescritas^(55, 70)^.

***Monitorear y llevar un registro***. La adaptación durante la transición hospital hogar implica estar atentos a la condición de salud, el efecto del tratamiento y la respuesta al cuidado, así como llevar un registro de la evolución y su manejo^(62)^. Esto incluye monitorear los síntomas y manejos durante las actividades diarias, y saber cuándo y cómo reportar cambios^(51, 58, 75)^.

### Casos que Ilustran el Concepto

***Caso modelo***. La señora Anita es una mujer viuda de 88 años con funcionalidad y cognición intactas, que vivía con su hija. Anita ingresó al hospital por una fractura de tobillo y después del alta, regresó a casa. Su hija se mostró dispuesta a afrontar la situación y asumir su cuidado (afrontar). La hija pidió a los profesionales de la salud que le explicaran la condición de su madre y cómo debía cuidarla, lo que le permitió comprender bien la situación (discernir). Luego recibió apoyo de enfermería mediante instrucciones escritas y verbales durante el alta hospitalaria (apoyar). Durante tres semanas, tuvo que mantener la pierna afectada elevada para evitar la hinchazón (prevenir) y cuidar la herida dos veces al día, atendiendo así las instrucciones que el médico le dio (transferir). Después de 20 días, Anita inició su rehabilitación y comenzó a reorganizar las cosas junto con su hija, mientras se adhería a las indicaciones y ejercicios para la curación. Luego continuaron los tratamientos farmacológicos habituales y los ejercicios de rehabilitación según lo recomendado (adherirse). Al ir a la cita de control, Anita y su hija presentaron los registros que habían diligenciado sobre los ejercicios realizados y la presión arterial semanal (monitorear y registrar). Estos registros permitieron hacer un pequeño ajuste en su medicación. Hoy Anita y su hija han retomado sus rutinas habituales, las cosas parecen volver a la normalidad aunque desde su accidente aprendieron a estar más atentas a prevenir situaciones que le puedan generar nuevas caídas.

***Caso límite.*** Camilo y María tienen 43 y 40 años respectivamente, llevan 10 años casados y decidieron no tener hijos. Ellos sienten que su apoyo mutuo ha sido definitivo para superar las dificultades. Camilo salió del hospital tras iniciar su tratamiento de hemodiálisis y tendrá que volver dos veces por semana por el resto de su vida o acudir al programa de diálisis peritoneal hasta que le encuentren un donante. El personal sanitario le ha explicado que esta situación no cambiará y aunque Camilo lo entiende, María no cree lo que le dicen; ella espera que la función renal de Camilo se restablezca para que pueda continuar con sus actividades diarias. Ella dice conocer casos de problemas urinarios que han mejorado solos. Contrataron un servicio de urgencias para cualquier imprevisto basándose en el miedo a la situación. María y Camilo siguen las instrucciones de cuidado de alimentos en la preparación del menú diario, salvo pequeños bocados que no incluyen en el registro de alimentación que deben llevar al control médico “para que el personal de salud no nos regañe”.

***Caso contrario***. Juan es un mensajero que presentaba dolores abdominales. Cuenta con seguridad social subsidiada por el Estado y vive con Marta, su esposa y sus hijos gemelos de un año. Acudió a urgencias y le dijeron que tenía apendicitis y que necesitaba una cirugía urgente. Marta pidió ayuda a una vecina para cuidar a sus hijos mientras ella ayuda a Juan en el hospital, pero hubo complicaciones y Juan tuvo que quedarse solo en el servicio de cirugía durante días. Marta solo podía entrar en horario de visita, pero en ese espacio la vecina no podía ayudarla a cuidar a los gemelos. Juan dijo que lo abandonó y quiere huir. No sabe cómo manejar la situación. No pidió apoyo en el hospital porque pensó que no se lo darían. Finalmente, Juan salió del hospital y omitió las instrucciones para su cuidado, como tomar medicamentos y descansar; él no sabía cómo reclamar sus medicinas. Ocho días después, tuvo que ser ingresado nuevamente por infección. La situación que es abrumadora para Juan y Marta ha cambiado significativamente su relación.

### Identificación de Antecedentes y Consecuencias

***Antecedentes***. La adaptación durante la transición hospital - hogar de la diada paciente – cuidador familiar exige que ellos estén listos para el alta, que tengan orientación y apoyo durante este periodo, que comprendan y se involucren en el cuidado, resolviendo sus dudas y garantizándoles un seguimiento^(63, 65, 68)^. Esta adaptación demanda que ellos se sientan reconocidos y respetados como personas y que se propicie el fortalecimiento de sus capacidades de cuidado mirándolos integralmente^(76, 77)^. Son de especial relevancia en este proceso la información, comunicación y coordinación adecuadas antes del egreso hospitalario^(61, 67, 70, 78, 79, 80, 81)^; así como la revisión y ajuste en las condiciones del contexto y la cobertura de las necesidades de atención en salud^(69, 82)^.

Es necesario que esta diada reconozca su condición y sus capacidades para afrontar con éxito el reto del cuidado en medio del desconocimiento, el temor y la vulnerabilidad pero con el deseo de seguir adelante buscando disminuir la carga y lograr bienestar en la vida diaria^(49, 56, 64, 70, 71, 74, 83, 84)^. Implica que la diada pueda satisfacer las necesidades de cuidado durante el proceso para enfrentar las barreras y aprovechar las oportunidades^(72, 85)^. Requiere a los implicados buscar el control y un estado de armonía para poder aprender el manejo de una nueva condición de salud que se suma a las demandas habituales^(16, 46, 50, 55, 65, 86)^. Supone que ellos generen estrategias de afrontamiento positivo que les ayuden a percibir su capacidad para cuidar, a disminuir el estrés o ansiedad, a fortalecer su potencial, sus recursos y su vínculo mutuo y a reconocer sus limitaciones y aumentar su nivel de resiliencia^(34, 38, 52, 54, 57, 62, 63, 74, 78, 84, 87, 88, 89, 90, 91)^. Para su afrontamiento la diada requiere manejar el conflicto y abordar el duelo^(49)^, así como contar con la motivación y orientación desde el hospital para asumir el cuidado en el hogar ^(50,59.60,63,66,68,69,84,85)^.

Lograr el discernimiento requerido para poder comprender y diferenciar la situación y los cuidados necesarios, es un proceso que toma tiempo y que orientación y educación específica frente a las tareas de cuidado, mientras se logra crecer con la experiencia^(34, 38, 45, 46, 58, 62, 70, 72, 73)^.

Lograr el apoyo efectivo requiere que la diada lo acepte como algo útil y confiable^(44, 73, 87)^. Los profesionales e instituciones por su parte, deben reconocer a las personas en su situación actual, orientarlas de manera adecuada, coordinar los procesos y servicios, explicarles en detalle el plan de cuidado, ratificar el buen desempeño y motivarlos a involucrarse para lograr los mejores resultados posibles durante la transición hospital hogar^(16, 34, 37, 39, 40, 41, 42, 44, 45, 46, 49, 50, 51, 52, 53, 57, 58, 59, 60, 62, 63, 67, 77, 90, 91, 92, 93)^.

Prevenir exige conocer de forma anticipada las necesidades o problemas que pueden presentarse y prepararse para resolverlos o para minimizar los riesgos durante y después de la transición^(40, 42, 45, 46, 71, 74, 92)^.

Al salir del hospital la diada requiere preparación y acompañamiento ya que recibe mayor responsabilidad sobre el cuidado y debe atender y aplicar las instrucciones recibidas^(45, 50, 53, 56, 58, 71)^. En tal sentido es necesario que los profesionales verifiquen tanto su nivel de comprensión como su habilidad para asumir esta responsabilidad antes del alta^(59, 64)^. Contar con pautas o protocolos clínicos facilita procesos de calidad y ayuda a prevenir complicaciones innecesarias^(16, 67)^.

La adherencia está influenciada por las realidades prácticas de mantener el tratamiento y por la disposición interior para hacerlo; lograr una buena adherencia implica que se cuente con independencia y credibilidad en quienes orientan el tratamiento y el cuidado para hacer lo que se debe y evitar lo que no se debe hacer^(90)^.

Finalmente el seguimiento a la condición de salud manteniendo un monitoreo constante con registro de los hallazgos relevantes es necesario para ayudar a la toma de decisiones y poder recibir mejor orientación para el manejo de la condición^(41,47,62,71).^

Un registro adecuado evita omisiones y facilita concretar las necesidades y apoyos requeridos durante los seguimientos profesionales^(64)^. El empleo de la tecnología, previa valoración de su pertinencia puede facilitarlos^(93, 94)^.

***Consecuencias.*** Quienes se adaptan durante la transición hospital - hogar logran retomar el control de la situación con mayor autonomía en el manejo de las tareas del cuidado. Ellos pueden lograr una nueva normalidad con satisfacción personal, confianza y comodidad al integrar el cuidado a la rutina diaria. Pueden vislumbrar el futuro a partir de la nueva condición y establecer un plan de vida en donde la preocupación y la ansiedad disminuyen. Finalmente pueden superar los obstáculos para integrarse a la propia comunidad^(44, 51, 54, 57, 59, 62, 71, 73, 75, 88, 90, 91, 92, 95)^.

La adaptación del paciente y su cuidador familiar durante la transición hospital hogar refleja un afrontamiento adecuado que genera confianza, autocontrol y autorregulación y permite organizar las rutinas y tareas del cuidado^(50, 51, 53, 55, 56, 71)^. Esto les genera una percepción positiva con sentido de competencia y control en medio de una rutina alterada^(16, 35, 40, 42, 53, 54, 56, 58, 59, 80)^.

De igual forma la adaptación se asocia con un buen nivel de discernimiento, lo que le permite a la diada comprender y poder manejar mejor la condición de salud que enfrenta, el diagnóstico y pronóstico, los signos y síntomas, el tratamiento, la medicación, los cuidados requeridos, las actividades recomendadas y el impacto de la situación de salud en su vida diaria^(37, 49, 70, 71, 74)^.

Una adaptación adecuada durante la transición hospital hogar del paciente y su cuidador familiar supone haber identificado o construido una red de apoyo que facilita la experiencia^(63, 74)^. Refleja que los profesionales e instituciones han dispuesto los recursos adecuados y que han hecho seguimiento a sus necesidades fluctuantes para responder a sus requerimientos^(49, 55, 70, 71, 85, 86)^.

Cuando las diadas se adaptan en este proceso es también evidente la existencia de un plan de cuidado en el cual se toman precauciones personales y ambientales y se identifican signos de emergencia o de alarma asociados con la condición de salud^(51, 57, 62)^. Prevenir les ayuda a mejorar la calidad de vida y les evita complicaciones, gastos innecesarios, reingresos hospitalarios y el uso innecesario de servicios especializados^(52)^.

Si bien las tareas o metas terapéuticas pueden ser inicialmente complejas, con el paso de los días estas llegan a convertirse en parte de una rutina diaria^(50, 65, 73)^. Sin embargo, para lograrlo es necesario que el paciente y su cuidador familiar pregunten y que se involucren en el cuidado tanto como sea posible^(43, 58)^. Es necesario además disponer los recursos, adaptaciones, equipos e información necesaria para garantizar en casa el cuidado requerido por estas personas^(52)^. Asumir la transferencia instrumentalizando las instrucciones de cuidado de manera adecuada permite a la diada desarrollar autonomía y aumentar el nivel de competencia^(73)^.

Adherirse ha demostrado ser una práctica que ayuda a superar la situación crítica de salud y generar un mejor pronóstico de vida^(41, 46, 59)^; ayuda además a disminuir riesgos y mejorar el efecto de los tratamientos que inician en el hospital y continúan en el hogar^(67)^. Un buen nivel de adherencia al tratamiento es una condición que refleja que hay un proceso de adaptación para el cuidado^(55)^.

De igual forma registrar permite observar de manera más objetiva la evolución y efecto del tratamiento y la adquisición de competencias para el cuidado en la diada, ayudando a precisar su nivel de adaptación durante la transición hospital hogar^(72)^.

El resultado de una adaptación positiva para el cuidado permite bajar el estrés percibido, mantener la mejor calidad de vida, mejorar el estado de ánimo y la satisfacción con el propio desempeño en el cuidado^(45, 52, 58, 87, 89, 93)^. Por el contrario, la falta de adaptación se asocia con mayores complicaciones y costos^(66)^ y aumenta la brecha entre la teoría y la práctica del cuidado de la salud^(47)^. Cuando esto sucede es necesario identificar lo que hace difícil la adaptación, lo que las personas quisieran que fuera diferente y aquello que les puede ayudar en su proceso^(60)^. La Figura 2 ilustra los antecedentes, atributos y consecuencias del concepto “adaptación durante la transición hospital-hogar” (ver Figura 2).

**Figura 2:** Adaptación del paciente y su cuidador familiar durante la transición hospital-hogar

Fuente, elaboración propia.

### Definición de Indicadores Empíricos

La adaptación de la diada paciente- cuidador familiar durante la transición hospital - hogar ha sido valorada de diferentes formas. La literatura reporta instrumentos aplicables a esta adaptación que evalúan competencias de cuidado en el hogar^(71, 96)^; calidad de la atención durante la transición van^(97, 98, 99, 100)^; adopción del rol de cuidador^(101)^; adoptar la capacidad de cuidar^(102)^; capacidad de afrontamiento^(103)^; preparación y alistamiento para el alta hospitalaria donde se revise además de la condición de salud el nivel de comprensión de la condición, la capacidad de afrontamiento y el nivel de apoyo disponible^(104, 105)^; la continuidad de la atención después del alta^(106).^ La prevención de complicaciones innecesarias como el manejo de medicamentos al regresar al hogar^(107)^; Algunas de estas herramientas se han validado culturalmente^(108)^ Sin embargo, algunos autores señalan que ninguno de estos indicadores aborda de manera integral la adaptación de la díada persona hospitalizada – cuidador familiar durante la transición hospital – hogar^(109)^. Una búsqueda sistemática reciente confirma estos hallazgos^(110, 111)^ (Ver Figura 3).

**Figura 3:** Indicadores empíricos de la adaptación del paciente y su cuidador familiar durante la transición hospital-hogar

Fuente, elaboración propia.

## DISCUSIÓN

El análisis del concepto “adaptación de la díada persona hospitalizada – cuidador familiar durante la transición hospital – hogar” es una contribución al conocimiento vigente. De una parte se suma con algunos conceptos relacionados con este tema como el concepto de transiciones para enfermería^(112)^; el concepto de cuidado centrado en el paciente con empoderamiento y participación en el cuidado hospitalario^(113)^; el concepto de cuidado centrado en la persona^(114)^; el de apoyo de pares en el contexto del cuidado de la salud^(115)^; y el concepto de cuidado transicional a nivel de los sistemas de salud^(116)^. De igual forma complementa los modelos y teorías relacionados con esta temática como son el modelo para el análisis de la adaptación a las transiciones humanas^(117)^ y los modelos de cuidado durante la transición^(118)^.

Desde la perspectiva teórica de la enfermería, el concepto de “adaptación del paciente y su cuidador familiar durante la transición hospital – hogar” es compatible con los postulados del Modelo de adaptación de Callista Roy y las teorías de mediano rango desarrolladas a partir de este, en donde se plantea cómo la enfermera, debe facilitar la adaptación del paciente para promover su salud y bienestar^(119, 120)^. Con respecto a la teoría de las transiciones propuesta por Meleis et al.^(121)^ que incluyen las que experimentan los individuos a lo largo de su vida, las que se dan entre la salud y la enfermedad, las que se presentan con la edad y aquellas que se asocian a otras situaciones de cambio, este concepto recoge, como lo señala su autora, el contexto, las emociones y las estrategias de afrontamiento del paciente y de su cuidador familiar ya que ellas pueden afectar el proceso adaptativo durante la transición^(122)^. El presente estudio ratifica los planteamientos de Ribeiro et al^(123)^, respecto al importante nivel de coherencia entre las concepciones teóricas de Callista Roy y las de Afaf Meleis, y su aplicación en la práctica profesional. El concepto analizado refleja que la adaptación es un fin y la transición, es el escenario cambiante donde se desarrolla el proceso de cuidado de la salud por parte de la díada paciente- cuidador familiar. Estos planteamientos reflejan el análisis presentado por Meleis quien propone la evolución de la enfermería desde teorías grandes a medianas para abordar situaciones más específicas de la práctica^(124)^.

Estos hallazgos pueden ser útiles para los formuladores de políticas y para profesionales de enfermería docentes o asistentes como base para el desarrollo de intervenciones basadas en evidencia para mejorar la evaluación, el diagnóstico y los resultados durante la transición hospitalaria y de alta de los pacientes y sus cuidadores familiares. La claridad conceptual demuestra, como se ha documentado ser un elemento esencial de la práctica de enfermería^(125)^. El presente análisis fue abordado con criterios incluyentes en geografía e idiomas. Sin embargo, sus hallazgos podrían tener limitaciones frente a algunas prácticas específicas de transición hospital – hogar aún no reportadas en la literatura.

## CONCLUSIÓN

La adaptación durante la transición hospital-hogar es un evento deseable que puede ocurrir en varios niveles y períodos. Estos comienzan cuando los pacientes en compañía de sus cuidadores familiares ingresan al hospital y duran hasta que ellos estén en la capacidad de cuidar su salud. La adaptación durante la transición hospital hogar es reflejo de las mejores decisiones para que los pacientes y los cuidadores mantengan o mejoren su calidad de vida en sus hogares.

Esta transición crea cambios y desafíos para los pacientes y sus cuidadores familiares y está mediada por la forma en que afrontan las situaciones, cómo disciernen la información de la que disponen, el apoyo con que cuentan, la prevención y anticipación del riesgo y emergencias, su capacidad para traducir las instrucciones terapéuticas en acciones, su adherencia a las instrucciones de cuidado y tratamiento y del seguimiento y registro para facilitar la toma de decisiones.

La adaptación durante la transición hospital – hogar de estas diadas supone un reto para la enfermería que al comprender tanto la vulnerabilidad como el potencial de cuidado que tienen los pacientes y sus cuidadores familiares podrá apoyar en el logro de una mejor calidad de vida con menores complicaciones y carga de cuidados.

La definición, atributos, antecedentes, consecuencias de la adaptación durante la transición hospital hogar obtenido en este estudio proporciona una base teórica para futuras investigaciones. Esta información puede utilizarse para evaluar la adaptación durante la transición hospital hogar, con el desarrollo de herramientas de evaluación y generar formación e intervenciones basadas en teoría.
